# Notes and News

**Published:** 1887-02-26

**Authors:** 


### Notes and News.

COLLECTED AND COMMUNICATED.

HER MAJESTY has been graciously pleased to allow the Children's Hospital at Hull to be called the "Victoria Hospital for Sick Children."

HIS ROYAL HIGHNESS THE DUKE OF CAMBRIDGE will preside at the annual dinner of the Royal National Hospital for Consumption, Ventnor, to be held on 19th April, at the Hotel Métropole. He has also consented to preside at the Central Meeting in connection with the Metropolitan Hospital Sunday Fund, to be held on Saturday, June 18th next, the last day of the Hospitals Week.

THE Beau Site Convalescent Home at Hastings, which was opened in August last, has already fifteen beds in constant occupation. This institution was founded through the munificence of the Misses Brisco, for convalescent patients residing within a ten-mile radius of Hastings.

MISS MIRRLEES has generously presented the sum of £500 to the Buchanan Homœopathic and Eye Hospital at St. Leonard's, for the purpose of buying the garden attached to the hospital.——Lady Jessel has given £2,000 to the North London Hospital for the perpetual endowment of a bed at that institution, to be called the "George Jessel Bed."

THE Committee of the National Dental Hospital, Great Portland Street, have decided to admit registered female medical students to the practice of the hospital.

AT the recent annual meeting of the Turner Memorial Hospital, a special vote of thanks was passed to Miss Baldwin, the late matron, for her generosity in returning the whole of her salary, either in cash or articles, for the benefit of the Institution.

THOUGH the season is now somewhat advanced, hospital and infirmary balls have still to be chronicled. A very pretty ball was recently held in aid of the Newark Hospital; and, thanks to the assiduous energy of Mr. Parker, of Carleton Hall, the subscription ball at the Carlisle County Hall, in aid of the Cumberland Infirmary, was a complete success.

DR. STUART has been appointed house-surgeon to the Chorley Dispensary.

THE building fund of the new branch of the Brighton, Hove, and Preston Dispensary now amounts to over £1,200.

LAST year was financially speaking the best which the Charing Cross Hospital has yet experienced. The total income amounted to £29,129, the largest sum which has fallen to the share of Charing Cross Hospital since its foundation. The Council have been enabled to reduce the floating debt from £5,927 to £1,778, while a sum of £9,000 has been invested. An effort is to be made during the Jubilee year to raise a further sum of £20,000 for investment.

THE Essex Convalescent Home, which is housed at that newly discovered watering-place, Clacton-on-Sea, is not so well known as it should be. It is intended as a refuge for patients from all parts of the county of Essex, and for those of "London over the border." Last year 173 cases were admitted, all of whom returned home greatly benefited. Clacton is one of those happy places where acute rheumatism is said to be almost unknown.

THE Governors of the London Fever Hospital are to be congratulated on the low rate of mortality which last year's returns show. Out of 524 admissions, 501 were contagious cases; 492 patients were discharged cured, and 17 died, equivalent to a death-rate of only 3.3 per cent. Considering the usefulness of this hospital in minimising the spread of infectious disease, it certainly deserves a larger measure of support than it at present receives.

THE New Brighton Convalescent Institution is one of the best of its kind in the north of England. Liverpool naturally contributes the largest number of patients, but invalids come to it from all parts of Lancashire and Cheshire. Last year no less than 805 persons sought its benefits, an increase of 115 on the preceding year. More convalescent institutions are wanted throughout the country. The demand upon the resources of the New Brighton Convalescent Home is only on a par with that at other similar institutions.

LORD HALSBURY, at the Jubilee festival in aid of the National Hospital for the Paralysed and Epileptic, held at the Holborn Restaurant, ably and successfully pleaded for that most terrible form of suffering for which this institution provides. Including the Albany Memorial, this hospital has now a total of 180 beds, of which fifty-five have not yet been taken into use (despite very pressing applications), for the simple reason that the funds are altogether insufficient. The meeting was therefore virtually one for inaugurating a Jubilee Fund for the attainment of this object. Mr. Burford Rawlings, the secretary, announced at the close of the proceedings that the subscriptions to hand in connection with the festival amounted to £1,183.

THE following vacancies are announced this week, the amount of the salary being placed in each case after the name of the institution :—

**Honorary Medical Officer.**

Pimlico Dispensary. Attending Medical Officer.

**Resident Medical Officers.**

Dumfries Infirmary. Assistant House Surgeon. No salary.
Eccles Hospital, Manchester. Qualified House Surgeon.—£60.
Salford and Pendleton Hospital. District Surgeon. Doubly qualified.—£80.

**Non-resident Medical Officer.**

Worcester Medical Association. Assistant. Doubly qualified. —£130, and extras,

**Matrons and Superintendents.**

St. Mary's Hospital, Manchester. Matron.—£50.
Victoria Hospital, Burnley. Night Superintendent.—£28, with uniform.

**Trained Nurses.**

Burton-on-Trent Nursing Institution. One.
Brompton Consumption Hospital. One.
Kidderminster Children's Hospital. Charge Nurse.—£20, with uniform.
Manchester Royal Eye Hospital. One.—£24, with uniform.
Middlesbrough Infirmary. One.—£20, with uniform.
Oldham Infirmary. One.—£16, with uniform.
Shaw Street Hospital, Liverpool. Staff Nurse.

---

IN India, hospital extension is going hand-in-hand with the Jubilee celebration. The Nizam founds a Women's hospital, built at his own personal cost; a Women's ward is to be added to the Secunderabad Civil Hospital; while the Rajah of the little State of Kapurtalla has just laid the foundation stone of a new hospital to be called the "Victoria."

---

MR. J. S. MORGAN, whose munificence is well known, has made the noble offer of £10,000 to Guy's Hospital, provided that the amount—£100,000—for which the Governors recently appealed to the public, is raised by 1st May. At present the special fund amounts to rather less than £50,000. To secure Mr. Morgan's splendid offer it is necessary, therefore, to raise about another £40,000.

---

SOME six years ago, a poor man was the patient of the Kendal Hospital. He was too badly off to pay anything, so he was treated free. He was in due course cured, and left the hospital. Judge the Secretary's surprise recently, to receive a donation of £2 from the grateful patient, who has latterly come into a little money! Instances of such moral rectitude are so few and so far between that, when one occurs, it deserves to be placed on record.

---

THE French Hospital is to be rebuilt. Its present premises in Leicester Square, the lease of which expires in two years, are far too small for the annually increasing work of this useful institution. Recently the nineteenth annual dinner in aid of the funds was held at Willis's Rooms, when the chair was taken by M. le Comte d'Aubigny. Among the guests present was the Lord Mayor. The new hospital is to be built in Shaftesbury Avenue. Last year the in-patients at the French Hospital amounted to 3,871, and the out-patients to 9,052. The institution relieves sufferers speaking French without distinction as to nationality. The patients treated last year belonged to twenty different nationalities, chiefly French, Italians, Swiss, Germans, Belgians, and English.

THE annual meeting of the Governors of the Cancer Hospital at Brompton was held on Wednesday, the 16th instant, Mr. George T. Hertslet occupying the chair. From the report of the Committee, it appears that during the past year 1,028 new patients were received, 652 being in-patients, and 976 out-patients. The total number of visits of new and old out-patients was 6,276, against 5,360 in 1885. This continued increase in the work points to the necessity for increased funds, the reliable income of the charity being about £3,000 a year less than the ordinary expenditure, and an earnest appeal is made by the Committee for continued support, to enable them to carry on the important work of alleviating, and as far as possible arresting the growth of this terrible disease. The medical staff give a course of lectures, open to all medical practitioners and students, in the lecture-room of the hospital during the first three months of the year. The report and balance-sheet were unanimously adopted, and the usual vote of thanks accorded, after which the proceedings terminated.

---

MR. JOHN STONE, on his recent re-election to the presidency of the Bath Royal United Hospital, threw out the hint that this being the Jubilee year, the supporters of the institution might see their way to doubling their subscriptions. The hint is one which subscribers to hospitals generally might carry into practical effect. The fortunes of the old Bath Royal United Hospital are just now at a very low ebb, and it is much to be feared that unless the present year is more prosperous than the past, some of its beds—which now number a hundred—will have to be closed.

---

THE seventh of the present course of winter entertainments for in-patients of the National Hospital for the Paralysed and Epileptic, Queen Square, Bloomsbury, was given on Thursday last. The programme was provided by Mr. James Hallett, whose efforts were deservedly and greatly appreciated by all present. Mrs. St. Vincent Mercier will provide the next entertainment for the in-patients of the Markham Square, Chelsea Branch of the St. John's Hospital for Diseases of the Skin. The date fixed is 5th March.

---

UNION is strength. The force of this truth is well illustrated by what the Bethnal Green Working Men's Society have been able to accomplish on behalf of our hospitals and dispensaries. In 1859, the Victoria Park Hospital being in serious debt, a few working men of the locality banded themselves together, each agreeing to give a penny a week towards the funds of this institution. The operations of the Society are now no longer limited to one hospital. The Society has raised since its formation nearly £4,000 for the hospitals and dispensaries of East London, and at the present moment four of its members are life governors of the Victoria Park Hospital, ten of the Adelaide Dispensary, one of the London Hospital, and two of the Truss Society. All the members of the Society are entitled to patients' letters whenever they require them.

THE following sums have been recently received by the various Medical Charities, the name of the donor or the source from which they were obtained being given in each instance after the name of the Institution :—

**Donations.**

Dental Hospital of London, Leicester Square. Mr. H. B. Mildmay, £10. Mr. P. Reid, £10 10*s*. The Company of Leathersellers, £10 10*s*.
Hospital for Heart and Paralysis, Soho Square. The Company of Leathersellers, £10 10*s*.
Westminster Hospital. Messrs. T. and W. Farmiloe, £20. (Jubilee Offering.)
Royal General Dispensary, St. Bartholomew Close. The Governors of the Bank of England, £50.
East London Hospital for Children, Shadwell. The Company of Grocers, £75.
Metropolitan Convalescent Institution, Walton-on-Thames. Miss. E. Wynne, £10 10*s*.
East London Hospital for Children, Shadwell. Company of Leathersellers, twenty guineas.
National Hospital for Consumption, Ventnor. A further grant from the Trustees of the Prison Charities, per Mr. J. Watney.
Great Northern Central Hospital. Mr. Chas. H. de Soysa, £50; Company of Leathersellers, twenty guineas.
British Home for Incurables, Putney. Company of Leathersellers, twenty guineas.
Metropolitan Convalescent Institution, Walton-on-Thames. Mr. Finlay, £12 3*s*. 4*d*.

**Legacies.**

British Home for Incurables, Putney. Miss M. A. S. Cox, £100.
London Hospital. Mr. Samuel Isaac, £250.
South Charitable Infirmary, Cork. Mrs. Honner, £100.

---

FOR the first time in its career, the income of the District Infirmary, Heckmondwike, has more than balanced its expenditure. The operatives who so largely seek the benefits of this institution, increased their subscriptions last year by £141. We gather from the annual report, that during the past year 1,418 patients attended the infirmary, while 281 were visited at their own homes, involving 1,223 visits. The infirmary has now forty-five beds. A laundry, mortuary, and other offices are being built at a cost of £1,200.

---

THE funds of the Worcester Ophthalmic Hospital are not in a satisfactory state. In 1885, the income of the institution was £284 9*s*. 7*d*., while last year it had sunk to £229 13*s*. 11*d*. The donations, which were £50 in 1885, fell to £2 in 1886. The Worcester Ophthalmic Hospital is virtually a free hospital, the majority of the poor applicants being treated without tickets or charge of any kind. Last year the out-patients numbered 724, as against 663 in 1885.

---

THE first meeting of the present year of the delegates of the Hospital Saturday Fund was held at the Board Room, 41, Fleet Street, on Saturday evening, under the presidency of H. N. Hamilton-Hoare (Hon. Treasurer). Among the representatives of institutions present were: Vice-Admiral de Kautzon (Seamen's Hospital), Dr. S. Kennedy (St. Saviour's Hospital), Rev. N. Bromley (King's College Hospital). The certificates of 126 representative delegates were verified. The delegates proceeded to consider the best date for the Ladies' Collection, and it was resolved that the same should take place on June 25th. It was also resolved that a committee should be formed comprising representatives from the various local Committees, for the purpose of organising a series of meetings in every district, with a view to securing for the Jubilee year such an amount as shall be worthy of the working classes of London, and testify to their appreciation of the hospitals and dispensaries of the Metropolis. Invitations have been issued to the offices of the Hospital Saturday Movement in the provinces to attend a conference, to be held either in London or Birmingham, during the ensuing summer.

---

THE annual meeting of the Governors of the Peterborough Infirmary disclosed a happy state of affairs, and the existence of the best relations among all associated in the good work of this admirable institution. An *entente cordiale* is the very backbone of such an institution. The Peterborough Infirmary has acquired more than a local reputation, and with such an excellent matron and Nursing Association, it can hardly fail to continue upon its present successful path.

---

JUBILEE Hospital proposals proceed apace. In Blackpool the Jubilee Memorial is to take the form of a hospital and dispensary, with, if possible, a convalescent home.——The Maternity Hospital Jubilee scheme in Bristol is exciting a good deal of sharp controversy in the local press; the supporters and objectors appear to be pretty evenly divided.——An infectious hospital is one of the unsupplied wants of Chelmsford. It has been suggested that a portion of the local Jubilee fund might be employed for the provision of such an institution. A hospital tent outside the town is the only place to which patients can now be taken in the event of an epidemic occurring.——In Birmingham and its suburbs a great effort is to be put forth to raise £10,000 as a Jubilee fund in aid of the Queen's Hospital, which is now nearly £1,000 in debt. Last year no less than 28,262 patients attended this institution, an increase of over 2,000 on the number of the previous year.——The inhabitants of Skipton, in Yorkshire, have decided to celebrate the Jubilee year by the erection, at a cost of about £3,000, of a hospital for the treatment of infectious diseases.——A cottage hospital has been proposed for Leighton Buzzard as a local Jubilee memorial.—A proposal has been made to build a Jubilee cottage hospital for Huntley and the adjacent parishes, and a fund has been started to raise £2,000 for this purpose.——From the far north of Scotland, from the quaint little town of Forres, comes the news that the townsfolk are endeavouring to rear a Jubilee cottage hospital. Forres is desirous of emulating the example of Keith, and we hope the inhabitants will succeed. A cottage hospital, suitable for the place, can be built and equipped for about £4,000, and an effort is now being made to raise a moiety of this sum. ——A proposal has been made in Exmouth to celebrate the Jubilee by the erection of a new dispensary. Mr. A. B. Webber has offered £25, Miss Webber £10, and other friends of the institution have promised to support the proposal.——A new cottage hospital is to be erected for Ashburton and Buckfastleigh in commemoration of the Queen's Jubilee, the present little institution being now quite inadequate for existing requirements. The building fund already amounts to over £1,000. Mr. R. G. Abraham has offered an excellent site, half an acre in extent, on the Eastern Road. The building is to be commenced as early as possible.

